# Evaluating the Depth of Penetration of Calcium Hydroxide Mixed With Three Different Herbal Essential Oils Using a Confocal Laser Scanning Microscope

**DOI:** 10.7759/cureus.67414

**Published:** 2024-08-21

**Authors:** Charanya Chandrasekaran, Vandna James, Balagopal Sundaresan, Anisha Sebatni, Sarath Kumar, Venkat Vidya

**Affiliations:** 1 Conservative Dentistry and Endodontics, Chettinad Dental College and Research Institute, Chennai, IND; 2 Conservative Dentistry and Endodontics, Tagore Dental College and Hospital, Chennai, IND

**Keywords:** e. faecalis, depth of penetration, intracanal medicaments, thyme oil, basil oil, oregano oil

## Abstract

Introduction

The goal of endodontic therapy is to completely eliminate the infection and stop microbes from infecting or reinfecting the root canal and the periradicular tissues. Amongst the primary microorganisms, *Enterococcus faecalis *(*E. faecalis*), a Gram-positive anaerobe, is the main cause of pulpal and periapical inflammation causing root canal failure. Literature evidence shows that the gold-standard calcium hydroxide is ineffective against *E. faecalis *due to its resistance to the alkaline pH and proton pump mechanism. Herbal essential oils such as oregano, basil, and thyme are known to possess antimicrobial properties against *E. faecalis*. However, their combination with calcium hydroxide as an intracanal medicament and the depth of penetration is still unknown.

Aim

To evaluate the depth of penetration of calcium hydroxide mixed with three different herbal essential oils using a confocal laser scanning microscope.

Material and methods

Fifty single-rooted premolars were decoronated and randomly divided into five groups. Group 1 - Oregano oil with calcium hydroxide, Group 2 - Basil oil with Calcium hydroxide, Group 3 - Thyme oil with calcium hydroxide, Group 4 - Calcium hydroxide with saline, Group 5 - Negative control. The teeth were instrumented and inoculated with *E. faecalis *and incubated for 21 days. Calcium hydroxide mixed with respective oils or saline and 0.1% rhodamine B dye was placed in the canals and again incubated for 7 days. Two sections each of 1 mm were horizontally cut at 3 mm and 5 mm from the apex and later subjected to a confocal laser scanning microscope to evaluate the depth of penetration. One-way ANOVA, post-hoc Tukey test, and student t-test were performed.

Results

At the middle third, basil oil had the maximum depth of penetration (1377.47±14.1 µm) followed by oregano oil (1345.4±26.5 µm) and thyme oil (1160.4±24.6 µm). At apical third, basil oil (1152.4±31.6 µm) showed maximum depth of penetration, followed by thyme (988.3±26.2 µm) and oregano oils (419.5±19.8 µm). The depth of penetration of these oils was greater at the middle third than at the apical third.

Conclusion

Basil, oregano, and thyme oil have good penetration depth into the dentinal tubules and can be successfully used in root canal procedures as intracanal medicaments.

## Introduction

Inflammation of the pulp and periapical tissues is primarily caused by bacteria [1]. The goal of endodontic therapy is to completely eliminate the infection and stop microbes from infecting or reinfecting the root canal and the periradicular tissues [2]. Due to the complex nature of the root canal system, it must be mechanically prepared and disinfected in order to lower the number of microbial colonies there. Complete elimination of microbes from the root canal system is difficult to achieve, owing to its anatomical complexities and limited access to the instruments and irrigants used. In order to get rid of the remaining germs, it is therefore thought to be crucial to apply an intracanal medication having antimicrobial capabilities. However, the dentinal tubules are only partially permeable to conventional medications, which diminishes their antibacterial potential [1]. When a high pH is not maintained, calcium hydroxide, the most commonly used intracanal medication, has been demonstrated to be inefficient at eliminating *E. faecalis* [3]. Rocas et al. observed that *E. faecalis *was found to be nine times higher in failed root canal treatment cases [4]. Research shows that plant essential oils have been found to possess excellent pharmacological, antibacterial, and antifungal activity, and thus, are a promising source of new natural drugs [5]. Hammer et al found that oregano oil (*Origanum vulgare*) inhibited *E. faecalis* at ≤ 2.0(v/v) [6]. Also, thyme (*Thymus vulgaris*) and basil (*Ocimum basilicum*) oils are proven to possess anti-bacterial properties against *E. faecalis *[6,7]. Though we know the antimicrobial efficacy of these oils, the ability of these oils when mixed with calcium hydroxide and the depth of penetration of these oils into the canal when used as an intracanal medicament is unknown. The aim of the present study is to evaluate the depth of penetration of three herbal essential oils namely oregano oil, basil oil, and thyme oil with calcium hydroxide against *E. faecalis *using a confocal laser scanning microscope.

## Materials and methods

The study was conducted after approval from the institutional ethical board of Tagore Dental College and Hospital (Approval no. IEC/TDCH/154/2022). The sample size was calculated with a 95% confidence interval with p<0.05. Fifty single-rooted mandibular premolars that were extracted for orthodontic purposes were used for this study. Teeth with well-developed and non-fused roots were selected. Carious, restored, teeth with cracks or fractures, improper anatomy, and hypoplasia were excluded from the study. The premolars were decoronated to standardise the root length at 14 mm using a vernier caliper (Rabbit Force digital caliper, MM-DIGI-0150). The canals were instrumented up to the F3 Protaper Gold instrument (Dentsply Maillefer, Ballaigues, Switzerland). During canal preparation, irrigation was carried out using 2 mL of 3% NaOCl (Prime Dental Products Pvt. Ltd., Bhiwandi, India) and 2 mL of 17% ethylenediaminetetraacetic acid (EDTA) (Prevest DenPro Pvt Ltd, Gurugram, India) utilising a 30 gauge needle (Real Touch Enterprises, Pimpri-Chinchwad, India). The final rinse was carried out for 30 seconds with 2 mL of saline (Prime Dental Products Pvt. Ltd.). The teeth were dried using paper points (Diadent Group International, Chungcheongbuk-do, Korea) before being autoclaved for 20 minutes at 121°C under 15 psi. A 10 mL bacterial suspension of the *E. faecalis *ATCC 29212 strain (Sigma-Aldrich, Hamburg, Germany) was obtained after an overnight culture. The samples were then cultured for 21 days at 37°C and 100% humidity with *E. faecalis *from the brain heart infusion broth injected into the root canals. 

The samples were then randomly divided into five groups of 10 each, which are: Group 1 - oregano oil (Cyrus Enterprises Pvt. Ltd., Mumbai, India) with calcium hydroxide, Group 2 - basil oil (Cyrus Enterprises Pvt. Ltd., Mumbai, India) with calcium hydroxide, Group 3 - thyme oil (Cyrus Enterprises Pvt. Ltd., Mumbai, India) with calcium hydroxide, Group 4 - calcium hydroxide (Dentsply Maillefer, Ballaigues, Switzerland) with sodium chloride saline (0.9 % W/V) (Eurolife Healthcare Pvt. Ltd., Mumbai, India), and Group 5 - negative control (Table 1).

**Table 1:** Grouping of the medicaments.

Group	Item
Group 1	Oregano oil with calcium hydroxide
Group 2	Basil oil with Calcium hydroxide
Group 3	Thyme oil with Calcium hydroxide
Group 4	Calcium hydroxide with Sodium Chloride, Saline (0.9 % W/V)
Group 5	Negative control

A 0.2 gm of calcium hydroxide powder with 0.07 cc of oil/saline was taken as the standard powder-liquid ratio. A 0.1% rhodamine B (Sisco Research Laboratories Pvt. Ltd. (SRL), Mumbai, India) (0.01g of rhodamine B in 10 ml of deionized water) dye was added to the calcium hydroxide mixture using a sterile cement spatula on a dry sterile glass slab. The medicaments were placed inside the root canal using lentulospirals (Mani Inc., Tochigi, Japan), and the samples were incubated again at 37°C and 100% humidity for 7 days. 

The samples were then mounted vertically with the apex facing upward on acrylic blocks. Two markings were given, one at the apical third which was 3 mm from the apex, and one at the middle third, 5 mm from the apex. Horizontal cuts were made with Baincut LSS (Low Speed Saw) (Chennai Metco Pvt Ltd, Chennai, India) across these markings. The segments were standardized to a size of 1 mm. Continuous cooling was done using water to avoid friction due to heating during sectioning. 

The sections were visualised under a confocal laser scanning microscope (Stellaris 5, Leica Microsystems GmbH, Wetzlar, Germany) to evaluate medicament penetration depth and the software used was Leica Las X. For accurate visualization of all the images, sections were studied under 10X lens. A wavelength of 540 nm was used for absorption of rhodamine dye whereas 590 nm was used as emission wavelength. The fluorescence at 10X magnification was used to depict the penetration depth of the root canal irrigating solutions. To measure the depth of penetration, the division of the images was done into four different regions. The final penetration depth was the calculated from highest out of the obtained readings.

Statistical analysis was carried out by using, one-way ANOVA, post-hoc Tukey Test, and student t-test. The software used in the analysis was SPSS (Statistical Package for Social Sciences) Version 24.0 (IBM Corp., Armonk, USA). P<0.05 was considered as the level of significance.

## Results

**Figure 1:**
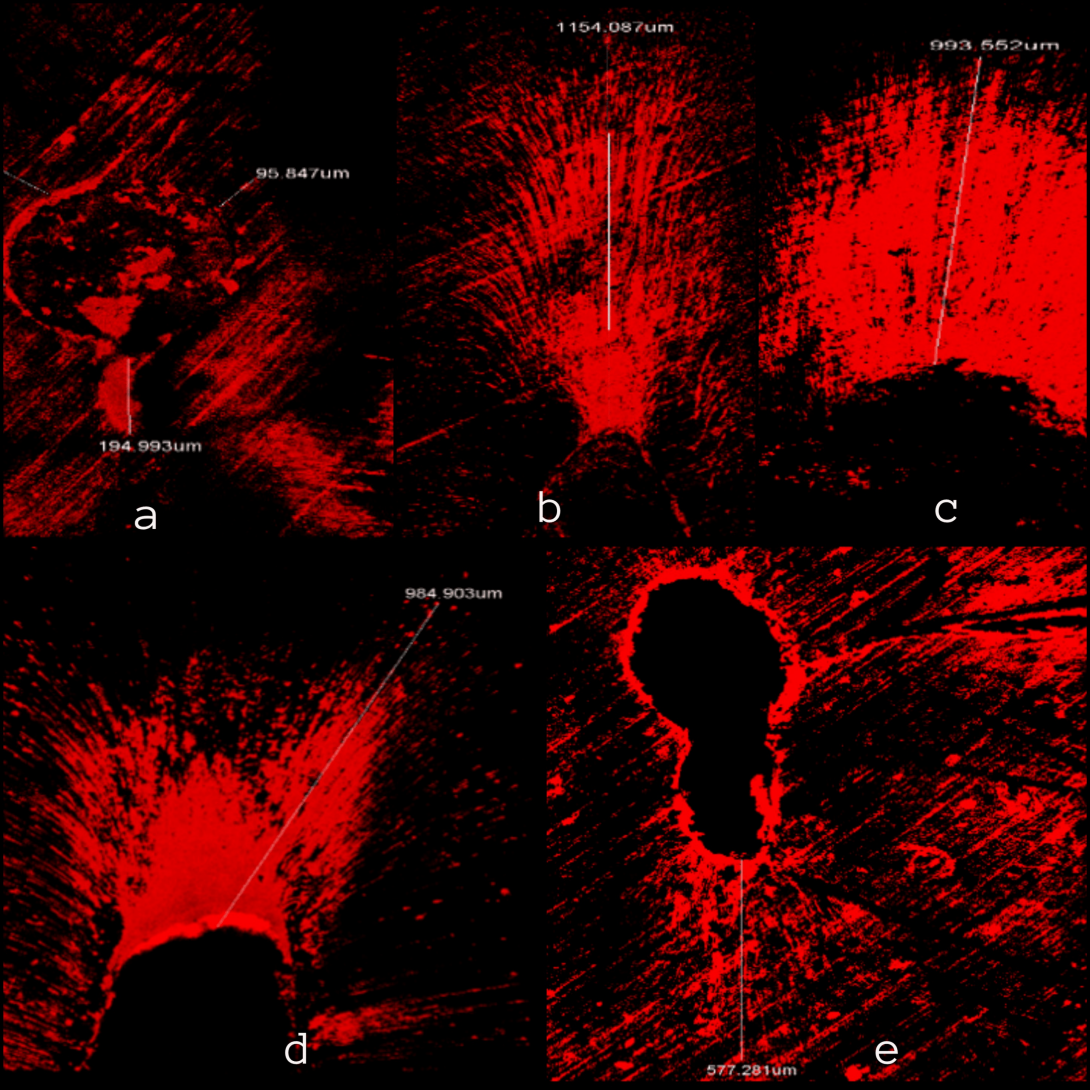
Depth of penetration at 3 mm from the apex a - Oregano oil with calcium hydroxide, b - Basil oil with calcium hydroxide, c - Thyme oil with calcium hydroxide, d - Calcium hydroxide with saline, and e - Negative control.

**Figure 2:**
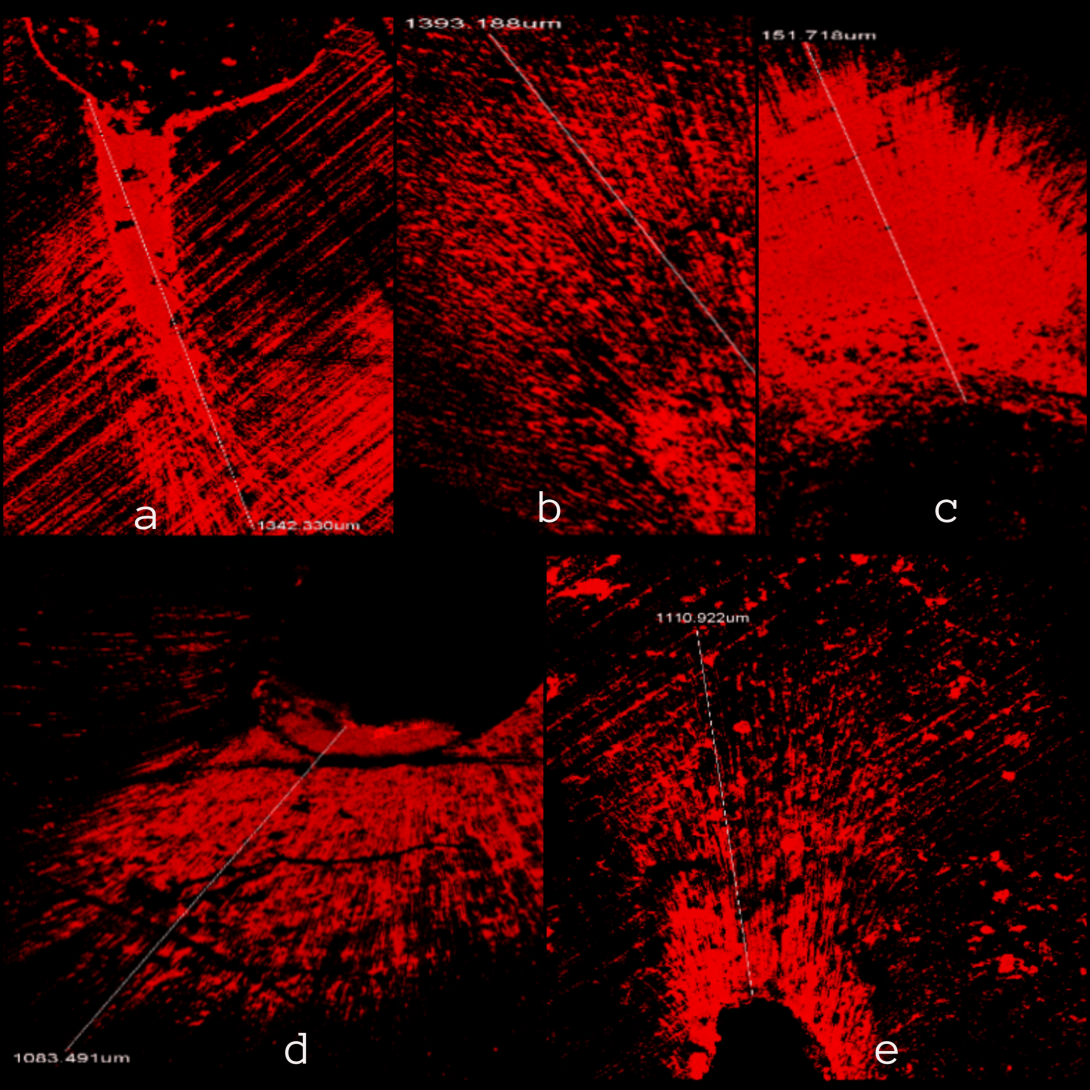
Depth of penetration at 5 mm from the apex a - Oregano oil with calcium hydroxide, b - Basil oil with calcium hydroxide, c - Thyme oil with calcium hydroxide, d - Calcium hydroxide with saline, and e - Negative control.

All sections were calculated for average depths of intracanal medicament penetration. A consistent fluorescent ring was seen around the canal in all sections (Figures 1, 2). An average value of 851.32±262.3 µm of medicament penetration was derived from apical sections whereas an average value of 1214.7±126.2 µm of medicament penetration was derived from mid-root sections, which was greater than apical sections.

At the middle third, basil oil had the maximum depth of penetration depth with a mean value of 1377.47±14.1 µm, followed by oregano oil with a depth of 1345.4±26.5 µm, and thyme oil, which had a mean value of 1160.4± 24.6 µm at 5 mm.

These values were greater than conventional calcium hydroxide and saline mixture. However, the results were not consistent with values obtained at the apical third. At the apical third, basil oil had the maximum depth of penetration of 1152.4± 31.6 µm, followed by thyme at 988.3±26.2 µm, and oregano oil with a mean penetration depth of 419.5± 19.8 µm. This might be due to the anatomical complexities encountered at the apical region (Table 2).

**Table 2:** Depth of intracanal medicament penetration SD: Standard Deviation, t: Paired t-test, p: Level of significance, F: Functional value. One-way ANOVA, Post-hoc Tukey test, and Student t-test were performed. * indicates statistical significance

Oils	Mean and SD		t	p
	3 mm from apex (Apical third)(In µm)	5 mm from apex (middle third)(In µm)		
Oregano oil with calcium hydroxide	419.5± 19.8	1345.4±26.5	-93.845	0.00
Basil oil with calcium hydroxide	1152.4± 31.6	1377.47±14.1	-21.067	0.00
Thyme oil with calcium hydroxide	988.3±26.2	1160.4± 24.6	-22.260	0.00
Saline with calcium hydroxide	987.2± 22.9	1077.9±23.7	-9.453	0.00
Negative control	709.0± 18.0	1112.6±26.3	-39.142	0.00
Total mean	851.32±262.3	1214.7±126.2	NA	NA
F	1428.104	341.227	NA	NA
P	0.00*	0.00*	NA	NA

## Discussion

*Enterococcus faecalis*, a Gram-positive anaerobic bacteria is the most common cause for failure in root canal treatment [8]. Although it only makes up a minor fraction of the flora in untreated canals, this persistent organism is a significant contributor to the development of chronic periradicular lesions after root canal treatment. It can exist in the canals either as a single organism or as a significant portion of the flora, and it is thus frequently discovered in a high percentage of root canal failures [9]. The resistant traits of *E. Faecalis *are its capacity for deep dentinal penetration, high pH tolerance, ability to endure conditions of food scarcity, and ability to survive independently without support from other microbial species [10]. The most used intracanal medication in endodontics is calcium hydroxide. In an aqueous solution, it separates into calcium and hydroxyl ions. The release of hydroxyl ions, which creates a strongly alkaline environment, is thought to be the cause of calcium hydroxide's antibacterial effect. The alkaline environment makes the majority of the bacteria in infected root canals incapable of surviving. Nevertheless, not all of the bacteria found in the root canal respond to it in the same way [10]. 

Essential oils are potent natural bioactive substances with established antibacterial activity. They are equally efficient against both multidrug-resistant and antibiotic-susceptible bacteria, even when arranged in biofilms. Depending on the amount of bioactive compounds present, the mechanism of action of essential oils can be complex; for instance, they may disrupt bacterial enzymes, respiration routes, membrane integrity, protein synthesis, or transmembrane transportation processes [11]. Being oil-based vehicles, plant essential oils can be employed as intracanal medicaments to maintain an alkaline pH for a longer period of time [12]. In a study done by Tiwari et al, where calcium hydroxide was mixed with oregano oil and rosewood (*Aniba rosaeodora*) oil as a new intracanal medicament, it was found to be effective against *E. faecalis *[5]. Herbal extracts with their superior properties like ease of availability, cost-effectiveness, low toxicity, anti-bacterial and anti-inflammatory effects are great alternative medicaments [13,14].

In this study, we have used a confocal laser scanning microscope to measure the depth of penetration of various intracanal medicaments. A confocal laser scanning microscope (CLSM) is an optical microscope that consists of laser light which acts as a light source and an electronic system which in turn processes the image. High-resolution optical images can be obtained in extremely thin sections (0.5 - 1.5 µm) and also eliminate the interference produced by different optical fields across the thickness of the sample [14]. In addition to enabling the viewing of materials with different compositions, CLSM gives appropriate information on the adaptation and/or dispersion of the materials throughout the root canal system and dentinal tubules. Also, as the CLSM permits the use of the same sample for additional analysis, it is known as a non-destructive approach [15]. Rhodamine B dye was utilised because of its smaller particle size, higher infusibility in dentinal tubules, and ease of viewing to see the penetration using CLSM [16].

In this study, it was found that the intracanal medicament mixtures had better penetration depth at the middle third than at the apical third. The intracanal medicament mixture with oregano oil had maximum penetration depth followed by basil at the middle third and all three of them had a better penetration depth when compared with calcium hydroxide mixed with saline. The difference in penetration depths at apical and middle thirds can be attributed to the anatomical complexities of the dentin which makes it difficult for the medicaments to penetrate deeper. At the middle third, due to the larger tubular size of the dentin and the absence of complex anatomical structures, there may be higher penetration depth [17]. In accordance with the above results, and also the fact that these herbal essential oils have already been found to be potent against *E. faecalis, *they can be used along with calcium hydroxide [5,6]. 

The limitations include further clinical studies with these potential herbal essential oils as an alternative to synthetic drugs against bacterial species such as *E. Faecalis *have to be evaluated. The alteration of the root dentin and the retrievability of these mixtures can be further studied for their long-term benefits.

## Conclusions

The three essential oils had a better penetration depth when they were mixed with calcium hydroxide at the middle third than at the apical third. Oregano oil, basil oil, and thyme oils mixed with calcium hydroxide had a greater depth of penetration when compared to calcium hydroxide mixed with saline. Basil oil showed a significantly greater depth of penetration at the middle and the apical third, proving its antibacterial efficacy against *E. Faecalis*. These essential oils, which have a potent antimicrobial efficacy against *E. faecalis, *can be used as alternatives to antibiotics, thus preventing root canal failures and contributing towards the success of evolving root canal procedures.
